# Petrogenesis of isotopically enriched Quaternary magma with adakitic affinity associated with subduction of old lithosphere beneath central Myanmar

**DOI:** 10.1038/s41598-022-07097-4

**Published:** 2022-02-24

**Authors:** Takashi Sano, Kenichiro Tani, Shigekazu Yoneda, Hla Min, Thaung Htike, Zin Maung Maung Thein, Osamu Ishizuka, Nao Kusuhashi, Reiko T. Kono, Masanaru Takai, Chris E. Conway

**Affiliations:** 1 Department of Geology and Paleontology, National Museum of Nature and Science, 4-1-1 Amakubo, Tsukuba, Ibaraki 305-0005 Japan; 2 Department of Science and Engineering, National Museum of Nature and Science, 4-1-1 Amakubo, Tsukuba, Ibaraki 305-0005 Japan; 3 Department of Geology, Meiktila University, Meiktila, Myanmar; 4 Pro-Rector, University of Magway, Magway, Myanmar; 5 Department of Geology, University of Mandalay, Mahaaungmyae, 05032 Mandalay, Myanmar; 6 Research Institute of Earthquake and Volcano Geology, AIST, 1-1-1 Higashi, Tsukuba, Ibaraki 305-8567 Japan; 7 Faculty of Science, Ehime University, 2-5 Bunkyo-cho, Matsuyama, Ehime 790-8577 Japan; 8 Faculty of Letters, Keio University, Hiyoshi 4-1-1, Yokohama, Kanagawa 223-8521 Japan; 9 Primate Research Institute, Kyoto University, Inuyama, Aichi 484-8506 Japan; 10 Japan Agency for Marine-Earth Science and Technology, 2-15 Natsushima, Yokosuka, Kanagawa 237-0061 Japan

**Keywords:** Geochemistry, Petrology, Volcanology

## Abstract

We present a model for the petrogenesis of magma with adakitic affinity in an old subduction zone, which does not involve slab melting and is constrained by new geochronological and geochemical data for Mt. Popa, the largest of three Quaternary volcanoes in central Myanmar (Popa, Monywa and Singu). The edifice is composed of Popa Plateau (0.8–0.6 Ma) with high-K rocks and a stratovolcano (< 0.33 Ma) predominantly composed of medium-K rocks with adakitic affinity (Mg# 45–63, Sr/Y > 40). The distinct K contents indicate that the adakitic magmas cannot be derived from Popa high-K rocks, but they share trace-element signatures and Sr–Nd isotope ratios with medium-K basalts from Monywa volcano. Our estimation of water contents in Popa magma reveals that primary magma for medium-K basalts was generated by partial melting of wedge mantle with normal potential temperature (T_P_ 1330–1340 °C) under wet conditions (H_2_O 0.25–0.54 wt%). Its melting was probably induced by asthenospheric upwelling that is recognized by tomographic images. Mafic adakitic magma (Mg# ~ 63, Sr/Y ~ 64) was derived from the medium-K basaltic magma in fractional crystallization of a garnet-bearing assemblage at high pressure, and felsic adakitic rocks (Mg# ~ 45, Sr/Y ~ 50) were produced by assimilation-fractional crystallization processes at mid-crustal depths.

## Introduction

**Figure 1:**
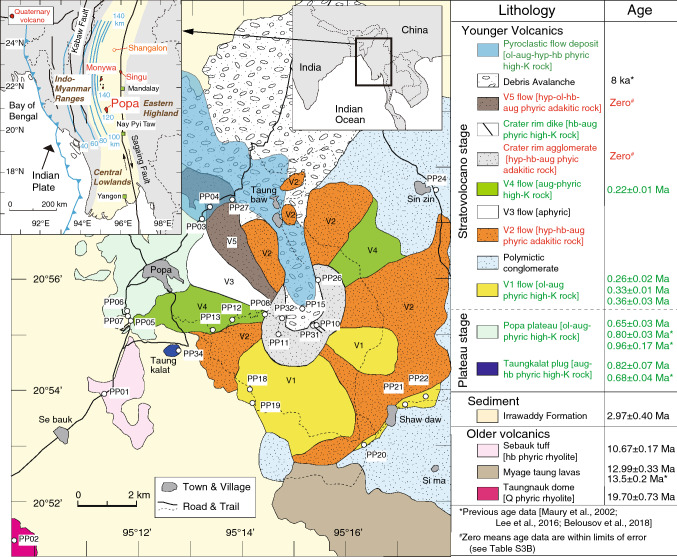
Geological map and stratigraphy of Popa volcano (modified after ref.^53^). Sampling locations and radiometric ages are also shown. Top-left inset shows locations of Quaternary volcanoes in central Myanmar with main tectonic features (simplified after ref.^17^) and depth contour lines of the upper surface of the subducting slab^11^. *ol* olivine, *aug* augite, *hyp* hypersthene, *hb* hornblende.

Myanmar lies at the junction of the Alpine-Himalayan orogenic belt and Indonesian Island arc system and is geologically divided into three major areas: Western Indo-Myanmar Ranges, Central Lowlands, and Eastern Highlands (Fig. 1). Three Quaternary volcanoes are present in the Central Lowlands; Popa, Monywa, and Singu (e.g., ref.^1^). They do not form a clear volcanic front, though Quaternary volcanoes can be traced southwards to volcanic islands in the Andaman Sea^2^. The slab-top depth beneath the volcanoes (> 120 km) is greater than the global average (~ 105 km; ref.^3^). Information about the construction history and magma origin for these volcanoes in this atypical setting is limited, and we report the first comprehensive geological dataset for Popa volcano, the largest of these volcanoes. New results from field observations, radiometric age determinations and geochemical analyses outline the first detailed growth history for this arc stratovolcano and clarify that this edifice is predominantly composed of Quaternary medium-K rocks with adakitic affinity.

Adakites are characterized by SiO_2_ ≥ 56 wt %, Al_2_O_3_ ≥ 15 wt %, MgO < 3 wt %, Na_2_O ≥ 3.5 wt %, Sr > 400 ppm, Y < 18 ppm, Yb < 1.8 ppm, Cr 30–50 ppm, Ni 20–40 ppm, Mg# (100 x [Mg/(Mg + Fe)] in mole) ~ 51, Sr/Y ≥ 40, La/Yb ≥ 20, K_2_O/Na_2_O ~ 0.5, FeO + MgO + MnO + TiO_2_ ~ 7 wt % and ^87^Sr/^86^Sr < 0.7040^4–7^ (see Supplementary Table S1). Such rocks were initially found in convergent margins associated with subduction of young (< 25 Ma) oceanic lithosphere^4^. Following ref.^8^, adakites have typically been interpreted as products of slab melting in young subduction settings, but several studies have challenged the viability of the slab melting hypothesis for adakitic or adakite-like rocks^9,10^.

Myanmar is presently located on a highly oblique subduction and slab tear arc setting^11,12^ that could be correlated with adakitic magmatism^13,14^. However, the slab beneath central Myanmar is composed of Cretaceous Indian and/or Neo-Tethyan oceanic crust and therefore the geothermal conditions do not support a slab melting origin for the magmas (e.g., ref.^15^). We integrate petrological and geochemical evidence to underpin a model for generating magma with adakitic affinity in an old subduction setting without slab melting, which is tested against recently published tomographic models for the region.

## Results

The products of Popa have been divided into ‘older volcanics’ and ‘younger volcanics’ based on the more extensive erosion that has affected the older volcanics^16^. Between these two stages, Pliocene sediments of the Irrawaddy Formation are present^17^. The main body of Popa is composed of the younger volcanics, which is subdivided into lower plateau and upper stratovolcano stages (Fig. 1). The plateau consists of Popa Plateau lava flows and the Taungkalat plug, and the stratovolcano is divided into 10 units mainly based on phenocryst assemblage characteristics (Fig. 1). We examined 31 samples from 3 units of the older volcanics, 3 scoria layers in the Irrawaddy Formation, and all volcanic units of the younger volcanics except for V3 flow and debris avalanche units (Fig. 1 and Supplementary Fig. S1). Two samples from the older volcanics are rhyolites with phenocrysts of quartz ± hornblende. The third sample from the older volcanics is andesite with plagioclase phenocrysts. In contrast, samples from the younger volcanics and scoria layers are basaltic to andesitic rocks with phenocrysts of augite ± olivine, hornblende, hypersthene, and plagioclase (Table S2). Micro-phenocrysts of apatite are present in andesites and zircons are included in all rocks (Supplementary Fig. S2). The mineral assemblages of all the andesitic rocks have common signatures (mineral assemblage of plagioclase, hydrous mineral, apatite and zircon) of adakites^4,5,18^, except for one sample (PP11) that has no hydrous mineral (Table S2).

The older volcanics were formed in the Miocene, as indicated by our U–Pb ages of zircon (19.7–10.7 Ma; Fig. 1 and Supplementary Fig. S3), which agree with previous U–Pb zircon age reports (13.5 ± 0.02 Ma; ref.^19^). A K–Ar age for air fall scoria (sample PP05A) in the Irrawaddy Formation indicates that activity of the younger volcanics began at least 3 Ma. For the plateau stage, our new age for Taungkalat plug (0.82 ± 0.07 Ma) is close to previous zircon U–Pb age constraints (0.68 ± 0.04 Ma; ref.^1^), but our Popa Plateau age (0.65 ± 0.03 Ma) is younger than previous reports (0.80–0.96 Ma; ref.^20^). This implies that Popa Plateau is composed of multiple lava flows with different ages. Our K–Ar and ^40^Ar/^39^Ar ages for sample PP19 are identical within analytical error, indicating that the beginning of the stratovolcano stage is ~ 0.33 Ma. A K–Ar age for sample PP21 from the same unit is younger (0.26 ± 0.02 Ma) than that of the lowest part, suggesting that the V1 flow unit is also composed of multiple lava flows. The younger ages of three upper units (0.22 ± 0.01 Ma for V4 flow, and Zero ages for Crater rim products and V5 flow) are consistent with the stratigraphy. See supplemental information for the age data (Table S3).

Mineral analyses show that olivine, two-pyroxenes, and hornblende phenocrysts have normal zoning with nearly uniform cores. In contrast, many plagioclase phenocrysts show oscillatory zoning consisting of high- and low-An cores/mantles (Supplementary Fig. S2; Table S4).

**Figure 2:**
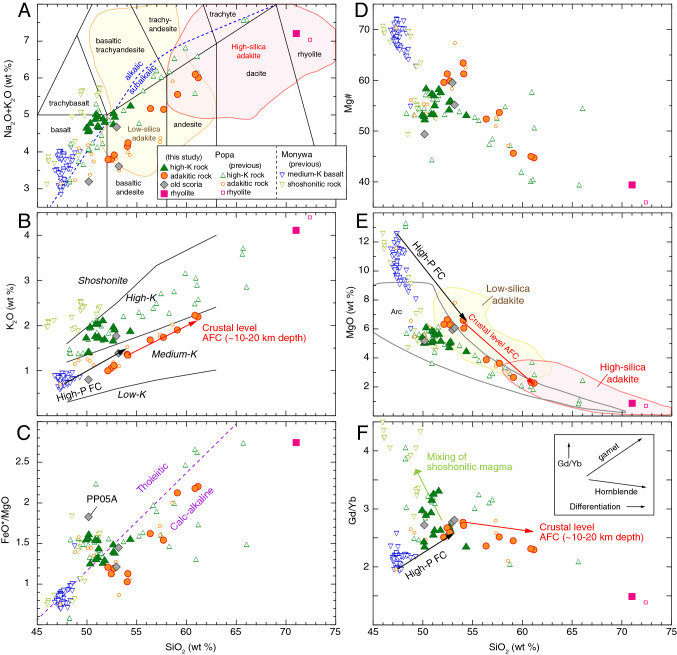
**(A)** Total alkali, **(B)** K_2_O, **(C)** FeO*/MgO, **(D)** Mg#, **(E)** MgO, and **(F)** Gd/Yb versus SiO_2_ diagrams for Quaternary volcanic rocks from Popa and Monywa volcanoes. Note that a highly altered sample (PP33; LOI ~ 10 wt%) is excluded. In **(B,E,F)**, black and red arrows indicate trends of high-pressure fractional crystallization (High-P FC) and AFC modeling (Tables S5, S6). In **(F)**, the green arrow indicates a proposed mixing line between adakitic and shoshonitic magmas to generate high-K magma. The two standard deviations of repeated sample analyses are generally less than the size of the symbols (Table S2). FeO*: Fe totals are reported as FeO wt%. Mg#: 100 × [Mg/(Mg + Fe)] in mole. Previous data for Popa and Monywa volcanoes are from refs.^1,16,19–21^. See the Supplemental Text for information of the rock classification and data fields.

**Figure 3:**
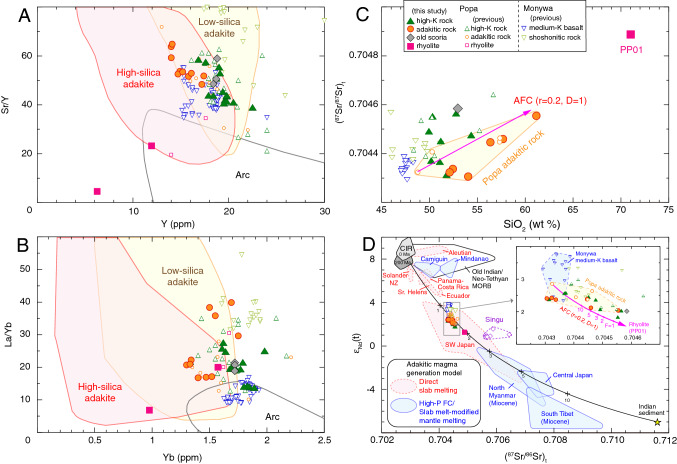
**(A)** Sr/Y versus Y, **(B)** La/Yb versus Yb, **(C)** initial (^87^Sr/^86^Sr)_t_ versus SiO_2_, and **(D)** initial ε_Nd_(t) versus (^87^Sr/^86^Sr)_t_ diagrams for the Quaternary volcanic rocks in central Myanmar. In **(D)**, black curved line with tick marks corresponds to percentage additions of pelagic sediment to Central Indian Ridge (CIR) magma source. In **(C)** and enlargement diagram in **(D)**, pink lines correspond to mixing of a rhyolitic magma (sample PP01) based on the open system AFC modeling calculated by using the equation in Table S6. D is the bulk distribution coefficient, F is the ratio of the initial to final magma mass, and r is the increment of assimilation and crystallization. The two standard deviations of repeated sample analyses are generally less than the size of the symbols (Table S2). Previous data for Popa, Monywa, and Singu volcanoes are from refs.^1,16,19–21^. See the Supplemental Text for information of the data fields and mixing calculation.

**Figure 4:**
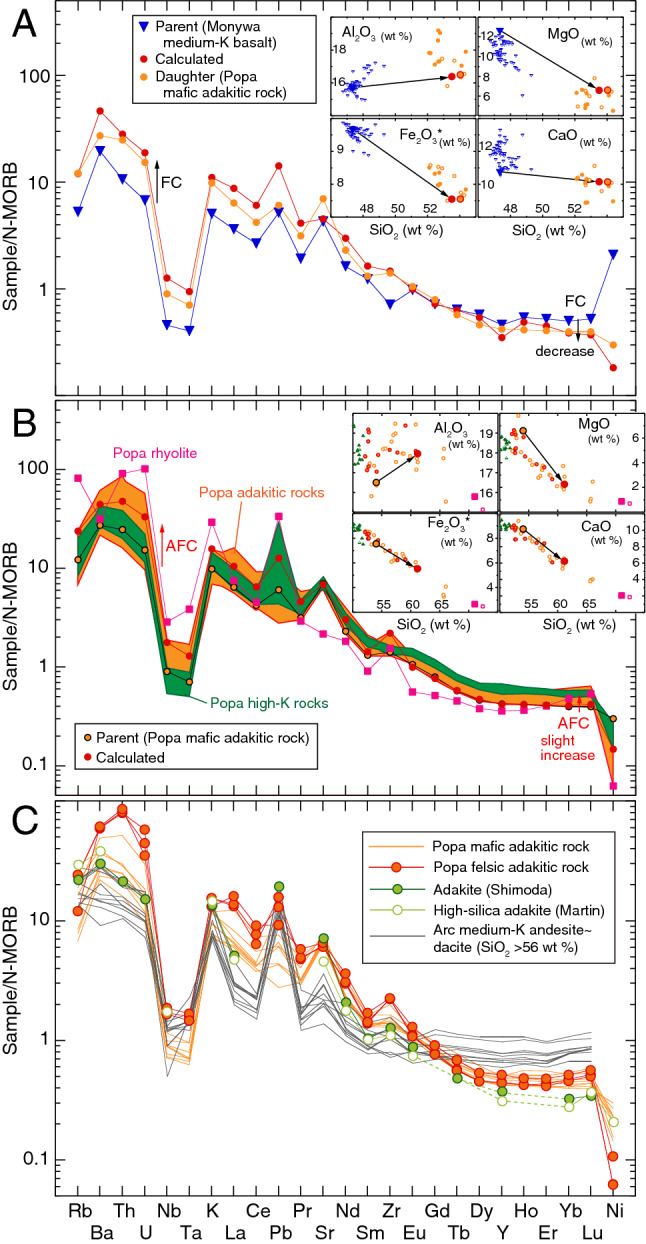
**(A,B)** Mass balance relationship between the parent and daughter samples from Popa and Monywa volcanoes, for both major element (insets on top-right) and trace element compositions (spider diagrams). **(A)** Monywa high-MgO medium-K basalt (sample 16MA02C) to Popa mafic adakitic rock (sample PP04) and **(B)** Popa mafic adakitic rock (sample PP04) to Popa felsic adakitic rock (sample PP08). See text for detailed explanation. Also shown are compositions of Monywa medium-K basalts and Popa volcanic rocks. Symbols in the major element diagrams and sources of previous data are the same as in Fig. 2. The two standard deviations of repeated sample analyses are generally less than the size of the symbols (Table S2). **(C)** Normal-MORB (N-MORB) normalized trace element diagrams for Popa adakitic rocks, a proposed adakite^25^, compiled high-silica adakite^18^, and normal arc medium-K andesites and dacites^33^. N-MORB data are from ref.^54^.

The rocks of the younger volcanics are classified into two types: high-K and adakitic rocks (Figs. 2, 3). The former plot in the alkalic field in Fig. 2A and high-K field in Fig. 2B. We define samples in the high-K field as high-K rocks and those in the shoshonite field as shoshonitic rocks, although the shoshonitic rocks are present for Monywa but not Popa volcano. The latter type are medium-K, calc-alkaline andesites with an affinity to the geochemical features of adakites (Figs. 2, 3 and Supplementary Fig. S4, Table S1) as explained below. Major element compositions of the adakitic rocks correspond to the low-silica adakite field (SiO_2_ < 58 wt %; MgO > 3 wt%), but three felsic adakitic rocks also plot in the high silica-adakite field (Figs. 2, 3). We classify “mafic adakitic rocks” as samples that plot only in the low-silica adakite field and “felsic adakitic rocks” for those in the high-silica adakite field. The mafic adakitic rocks have many geochemical features of adakites such as high Sr/Y (40–65), but are different from the low-silica adakites that have extremely high Cr (> 150 ppm) and Ni (> 100 ppm). Likewise, two mafic adakitic rocks fall slightly outside the field of Quaternary adakites on a La/Yb versus Yb diagram (Fig. 3). In contrast, the felsic adakitic rocks satisfy all the adakite definitions (e.g., Sr/Y > 48, La/Yb > 36, Sr > 773 ppm, Y < 17 ppm, Yb < 1.7 ppm, SiO_2_ > 59 wt%, MgO < 2.7 wt%) except for their lower Cr and Ni contents and their high ^87^Sr/^86^Sr isotopic ratios when compared with high-silica adakites (Figs. 2, 3, Supplementary Fig. S4; Table S1). Trace-element signatures of the felsic adakitic rocks are different from those of typical arc medium-K andesites and dacites (Fig. 4C), suggesting that Popa magma was not generated in a normal subduction zone. Instead, the felsic adakitic rocks have trace-element signatures with adakitic affinity. High-silica adakites are inferred to represent ‘pure’ slab melts unaffected by interaction with mantle wedge peridotite^7^. However, we propose Popa adakitic rocks were produced by differentiation of partial melt of hydrous and upwelling wedge (see below).

Popa stratovolcano consists of alternating layers of high-K and adakitic rocks (Fig. 1). Two old scoria samples in the sedimentary layers (Irrawaddy Formation) are also adakitic rocks (Figs. 2, 3), suggesting that adakitic rocks are predominant within the younger volcanics. In contrast, another old scoria plots in the field of tholeiitic basalt (PP05A in Fig. 2C), but its classification is not certain because of its moderate alteration (weight loss on ignition, LOI 3.75 wt%). Since the alteration could have affected the major element chemistry of the old (~ 3 Ma) scoria samples, a different symbol is used for the old scoria on our geochemical diagrams.

Popa adakitic rocks have more enriched isotopic compositions, with more radiogenic Sr and less radiogenic Nd isotope ratios than typical adakites (Aleutian, Solander and St. Helens) in Fig. 3D. The isotopically enriched signatures of Popa adakitic rocks are more easily identified by Nd isotopic ratios than Sr ones. We therefore focus on the Nd isotopic ratios to elucidate the origin of adakitic magma in “Discussion”.

The Sr–Nd isotopic ratios of Popa high-K rocks and adakitic rocks plot within a narrow field (Fig. 3D), implying that they originated from the same magma source. Minimal variability in the Sr isotopic ratios of adakitic rocks can be explained by an assimilation-fractional crystallization (AFC) trend, which extends to rhyolite sample PP01 (Fig. 3C,D). Notably, the least differentiated adakitic rocks, which have the lowest Sr isotopic ratios, plot within the compositional field of Monywa medium-K basalts^21^. Furthermore, Popa adakitic rocks share many trace-element signatures with Monywa medium-K basalts (Fig. 4).

## Discussion

### Genesis of Popa medium-K rocks with adakitic affinity

The evolved chemistry of Popa magmas with adakitic affinity (e.g., Ni, 6–30 ppm) indicates they were differentiated from a primary magma whilst traversing the arc lithosphere beneath central Myanmar. We conducted careful petrogenetic reconstructions here to constrain this evolution. Figure 2B shows that Monywa medium-K basalts, not Popa high-K rocks, are parental to the Popa adakitic rocks (i.e., medium-K rocks with adakitic affinity). To evaluate the parent-daughter relationships we have conducted fractional crystallization calculations via least-squares mixing calculations using major element compositions of whole-rocks and phenocryst minerals (insets on top-right of Fig. 4A; Table S5). We selected the highest Mg# Monywa basalt (sample 16MA02C; ref.^21^) as the parental liquid and the most mafic adakitic rock (sample PP04; Mg# 62, SiO_2_ 54 wt%) as the daughter liquid. In order to explain the higher Gd/Yb, Sr/Y and La/Yb of Popa adakitic rocks relative to Monywa medium-K basalts (Figs. 2, 3), we assumed high-pressure garnet fractionation (~ 1 GPa; e.g., ref.^22^). This pressure is consistent with an imaged low velocity zone at 30–40 km depth^23^, which provides supporting evidence for deep differentiation of Popa primary magmas. Averaged phenocryst mineral analyses (Table S4) were used in the calculation; for garnet and augite, we used compositions reported by high-pressure melting experiments^24^. We further examined the behavior of trace elements during fractional crystallization by using Rayleigh fractionation modeling (Table S6); the mafic adakitic rock can be produced by subtraction of the phenocryst phases (olivine, plagioclase, augite, hornblende) and garnet (Figs. 2, 4A). Notably, the subtraction of garnet in our calculation (1.2%) is lower than the residual garnet fraction of slab melting models (e.g., > 30%; ref.^25^). This result is consistent with the higher heavy rare-earth element contents of Popa adakitic rocks when compared with those of typical adakites (Fig. 4C).

Differentiation from the mafic adakitic rock (sample PP04; Mg# 62, SiO_2_ 54 wt%) to felsic adakitic rock (sample PP08; Mg# 45, SiO_2_ 61 wt %) can be explained by AFC, when accounting for assimilation of crustal material (Fig. 3C,D). Assuming that rhyolite of the older volcanics (sample PP01) is derived from partial melts of continental crust, we conducted least-squares mixing calculations using major elements (insets on top-right of Fig. 4B; Table S5) and AFC calculation using trace elements (Fig. 4B; Table S6). The results show that the compositional variation of Popa adakitic rocks is reproduced well by the AFC process.

Temperature and pressure (T–P) conditions of AFC processing were obtained from mineral geothermobarometers. Adjacent augite-hypersthene phenocryst pairs in two adakitic rocks and an averaged two-pyroxene phenocryst pair in sample PP08 provided crystallization temperatures of 880–942 °C (Table S4) using the geothermometer of ref.^26^. The result is within temperature ranges estimated using the geothermometer of ref.^27^ (Supplementary Fig. S5). Hornblende phenocrysts in four Popa samples yielded an average pressure of 410 ± 80 MPa (Table S4) using the Al-in-hornblende barometer of ref.^28^. This result is identical to that calculated by another barometer (460 ± 50 MPa; ref.^29^) as shown in Supplementary Fig. S6. The estimated pressures (~ 300 to 550 MPa) equate to depths of ~ 10–20 km in continental crust. Using the hygrometers of refs.^30^ and^31^, the H_2_O content of the adakitic magmas was estimated to be 3.6–5.5 wt% and 4.5–5.9 wt%, respectively (Supplementary Fig. S6; Table S4).

### Mantle melting conditions

**Figure 5:**
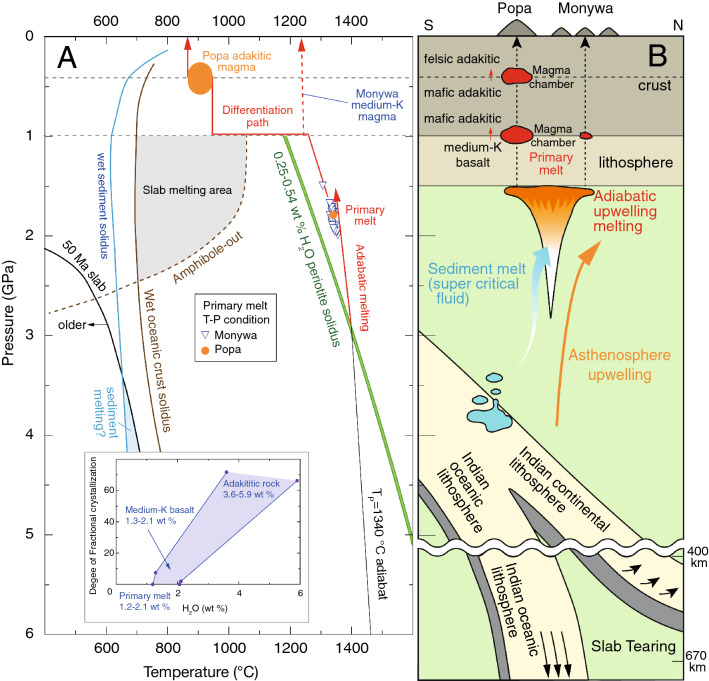
**(A)** T–P condition of the wedge mantle beneath central Myanmar, the estimated melting and differentiation paths to produce Popa adakitic rocks and Monywa medium-K basalts, 0.25–0.54% H_2_O peridotite solidus^55^, the hydrous oceanic crust and the stability field of amphibole^56^, hydrous sediment^57^, and the 50 Ma slab^14^. T_P_ is estimated to be ~ 1330–1340 °C, and adiabatic paths for solid (dT/dP = 20 °C/GPa) and melting (dT/dP = 34 °C/GPa) peridotite are after ref.^58^. Note that slab melting does not occur beneath old (> 50 Ma) and cold subduction zones. Inset in bottom-left shows the estimated degree of fractional crystallization versus H_2_O content of Popa adakitic, Monywa medium-K basaltic, and primary melts. **(B)** Schematic cross-section beneath central Myanmar to explain magma genesis at Popa and Monywa volcanoes.

We consider that Popa adakitic rocks and Monywa medium-K rocks were derived from the same primary magma beneath central Myanmar because they share near-identical trace-element signatures and Sr–Nd isotope ratios. Furthermore, the above fractional crystallization calculation suggests that Monywa medium-K magma is parental to Popa adakitic magma. By establishing the link between Popa adakitic rocks and Monywa medium-K basalts, crucial constraints can be placed on the nature of the mantle wedge beneath the Indochinese Peninsula. We estimated the H_2_O content in primary melts (1.2–2.1 wt%; Fig. 5) by using the subtracted mineral fractions from the fractional crystallization model. Further, H_2_O content in wedge mantle was estimated to be 0.25–0.54 wt % using the batch melting equation^32^ by considering the proposed melting degree of primary magma beneath a frontal arc (20–25%; ref.^33^). Following the method of ref.^21^, the T-P conditions of primary melt generation were estimated to be 1307–1356 °C and 1.5–2.0 GPa (see “Methods”). The estimated pressure is relatively higher than arc magma generation pressures beneath volcanic fronts (e.g., 1.0–1.5 GPa; refs.^33,34^). Such melting conditions would not be reached in a steady state subduction zone but can be achieved by adiabatic upwelling of deeper wedge mantle with normal potential temperature (Fig. 5; T_P_ 1330–1340 °C; ref.^35^). Seismological tomographic images reveal a zone of upwelling mantle in the wedge beneath central Myanmar^23^. This upwelling is probably related to the magma origin of Popa and Monywa volcanoes (Fig. 5B). One proposed trigger of the upwelling is slab tearing that extends from upper mantle (~ 150 km) to depths between 410 and 660 km^12^.

The slab tearing also induced a flow of hot plume beneath central Myanmar. Singu magma, which has ocean island basalt (OIB) trace-element and Sr–Nd isotopic signatures (e.g., Fig. 3D), was proposed to be formed by partial melting of the plume^19^.

Since the slab beneath central Myanmar is old (> 50 Ma in Fig. 5), much of the wedge mantle H_2_O was derived not by slab melting but dehydration of hydrous oceanic crust and the underlying peridotite slab (e.g., ref.^36^). However, at high pressures (> 3.5 GPa in Fig. 5), sediment melt (supercritical fluid at such pressures; ref.^37^) can originate in wedge mantle, even in old (> 100 Ma) subduction zone settings^38^. Sr–Nd isotopic ratios show that the magma source of Popa and Monywa has 1–2 wt % of sediment melt, and that Popa adakitic rocks and high-K rocks, and Monywa medium-K basalts, plot in the same field (Fig. 3D). We suggest that the Popa high-K rocks formed by mixing between adakitic magma and Monywa shoshonitic magma (Fig. 2F), which was produced by low-degree partial melting of the same hydrous mantle source that is also slightly contaminated by sediment melts^21^.

Previous studies showed that slab fluids played a key role in the genesis of adakitic magma in the double plate subduction and rear arc settings of central and northwest Japan, respectively^39,40^. Their interpretations were supported by detections of possible slab-derived fluids in wedge mantle based on high-resolution tomographic models^41,42^. For Myanmar’s old subduction zone setting, the processes of slab dehydration, adiabatic melting of wedge mantle and high-pressure fractionation identified in our petrogenetic reconstruction are independently verified by present-day tomographic models for the crust and mantle beneath Myanmar^23^. This multidisciplinary match-up indicates that (1) a plausible model for the generation of adakitic rocks caused by mantle upwelling can be demonstrated without slab melting, (2) mantle melt production and differentiation in transcrustal magmatic systems is ongoing beneath Popa and may fuel future adakitic volcanism via the outlined model.

The slab beneath central Myanmar is Cretaceous Indian and/or Neo-Tethyan oceanic crust, the Nd isotopic ratio of which is distinctly higher than that of the Popa adakitic rocks (Fig. 3D). If slab melts were the source of Popa’s felsic adakitic rocks (i.e., assumed to be ‘pure’ adakite unaffected by interaction with mantle wedge peridotite; ref.^7^), the Nd isotopic ratios of Popa rocks would be higher than what has been measured in this study. Further, lower Cr and Ni contents in Popa felsic adakitic rocks than those in high-silica adakites (Fig. 4C; Supplementary Table S1) indicate fractional crystallization of olivine and augite has occurred at crustal levels. In contrast, true adakites are rich in Cr and Ni because they are ‘pure’ slab melts with little modification by fractional crystallization (e.g., ref.^7^). The distinctly lower Cr and Ni contents of Popa mafic adakitic rocks than those of low-silica adakites (Supplementary Table S1) can be explained similarly. Cr and Ni contents in Popa mafic adakitic magma were significantly lowered by fractional crystallization of Monywa medium-K magmas (Fig. 4A), but those in low-silica adakites are high because they were likely produced by interactions between slab melts and the peridotite mantle. We therefore rule out the role of slab melting in the magma genesis of adakitic rocks for central Myanmar volcanoes.

## Conclusions

This study has fused evidence from petrology, major and trace-element modelling, isotopic systematics and geophysics into a robust model for generating medium-K magma with adakitic affinity in an old subduction zone setting (Fig. 5), which is summarized as follows. Adiabatic upwelling of normal T_P_ (~ 1330 to 1340 °C) wedge mantle beneath central Myanmar formed primary melt of Monywa high-K basalt at 1.5–2.0 GPa and 1.2–2.1 wt % H_2_O. This model suggests that wedge mantle beneath central Myanmar does not correspond to a steady state subduction zone but has an extra source of heat from the deep mantle wedge caused by adiabatic upwelling. Mafic adakitic magma (Mg# ~ 62, SiO_2_ ~ 54 wt %) from Popa volcano was produced by high-pressure (~ 1 GPa) fractional crystallization of Monywa medium-K basalts. Felsic adakitic rocks (Mg# ~ 45, SiO_2_ ~ 61 wt %) from Popa volcano were generated by AFC processing of the mafic adakitic magma at crustal levels (~ 10–20 km) at 880–942 °C and 4.5–5.9 wt% H_2_O. The isotopically enriched signature and low Cr and Ni contents in the Popa rocks with adakitic affinity indicate that a slab melting origin is not appropriate for these magmas. The further implication of these findings is significant; mantle melt production and differentiation is ongoing near the northern terminus of east-dipping subduction in western Southeast Asia, despite slow and highly oblique subduction.

## Methods

### Modal composition determination

Modal compositions of 28 basalts and andesites (Table S2) were determined at the National Museum of Nature and Science (NMNS) in Japan. Firstly, full-page, whole scanned images at a high resolution (3200 dpi) were prepared from thin sections by placing them on a standard scanner. Next, the scanned images were printed out, and outlines of different phenocrysts were traced using appropriate marker pens with different colors. During the traces, types of phenocrysts were checked by microscope images of thin sections. The traced sheets were scanned at high resolution once again, and areas of the different phenocrysts and groundmass were detected by a computer software (GIMP).

### Major and trace element chemistry

Major and selected trace elements in rock powders of 31 samples were determined using a Rigaku ZSX Primus II X-ray fluorescence (XRF) spectrometer at the NMNS. The sample preparation and XRF techniques followed the method of ref.^43^. The standard deviations (1σ) of calibration lines for major and selected trace elements are given in ref.^43^.

After XRF analysis, the same rock powders of 31 samples were used for analysis of a larger range of trace elements by inductively coupled plasma-source mass spectrometry (ICP-MS). Trace element compositions were determined using a quadrupole Agilent 7700 × ICP-MS instrument at NMNS and the procedures described by ref.^44^. Prior to ICP-MS analysis, the samples were digested using a HF-HClO_4_-HNO_3_ acid attack with final dissolution in 2% HNO_3_ plus 0.1% HF solution and spiked with ^115^In and ^209^Bi. These elements were added to standardize the signal for the ICP-MS measurements. Internal precision and external reproducibility are typically better than 1% and 3%, respectively. Because the Zr and Hf contents obtained by ICP-MS are expected to be low because of the presence of refractory zircon minerals in the sample solutions for the analyses (Supplementary Fig. S2D), we did not use these contents in this paper.

### Isotope ratio measurements

Among the samples analyzed for major and trace elements, 16 samples were selected for isotope ratio measurements (Table S2). Chemical separation of Sr and Nd were conducted at NMNS following the methods in the Supplementary Information. Sr and Nd isotope ratios were measured by thermal ionization mass spectrometry (TIMS: Thermo Fisher Scientific TRITON plus) at NMNS following the procedure described by ref.^45^. The measured Sr and Nd isotope ratios were normalized to ^86^Sr/^88^Sr = 0.1194 and ^146^Nd/^144^Nd = 0.7219, respectively, to correct for mass fractionation. The mean ^87^Sr/^86^Sr value of the NIST 987 was 0.7102412 (± 0.0000029; 2SD, n = 10) and the mean ^143^Nd/^144^Nd value of the JNdi-1 standard was 0.5121002 (± 0.0000016; 2SD, n = 10). The ^87^Sr/^86^Sr and ^143^Nd/^144^Nd of the samples are reported relative to NIST 987 or JNdi-1.

### ^40^Ar/^39^Ar dating

^40^Ar/^39^Ar ages (Table S3A) were determined using the ^40^Ar/^39^Ar dating facility at the Geological Survey of Japan (GSJ, AIST). Details of the procedures are reported by ref.^46^. Aliquots of 20–25 mg of phenocryst-free groundmass, crushed and sieved to 250–500 μm in size, were analyzed using a stepwise heating procedure. The samples were treated in 3 N HCl for 30 min with ultrasonic bath to remove any weathered material present. After this treatment, samples were examined under a microscope, and any remaining phenocrysts were removed. Sample irradiation was done at the CLICIT facility of the Oregon State University TRIGA reactor for 4 h. Sanidine separated from the Fish Canyon Tuff (FC3) was used for the flux monitor and assigned an age of 27.5 Ma, which has been determined against the primary standard for the GSJ K–Ar laboratory, Sori biotite, whose age is 91.2 Ma^47^. A CO_2_ laser heating system (NEWWAVE MIR10-30) was used at continuous wave mode for sample heating. A faceted lens was used to obtain a 3.2 mm-diameter beam with homogenous energy distribution to ensure uniform heating of the samples during stepwise heating analysis. Argon isotopes were measured on an IsotopX NGX noble gas mass spectrometer fitted with a Hamamatsu Photonics R4146 secondary electron multiplier in peak-jumping mode. Correction for interfering isotopes was achieved by analyses of CaF_2_ and KFeSiO_4_ glasses irradiated with the samples. The blank of the system including the mass spectrometer and the extraction line was 2.9 × 10^−14^ ml STP for ^36^Ar, 1.4 × 10^−13^ ml STP for ^37^Ar, 1.0 × 10^−14^ ml STP for ^38^Ar, 1.2 × 10^−14^ ml STP for ^39^Ar and 1.9 × 10^−12^ ml STP for ^40^Ar. The blank analysis was done every 2 or 3 step analyses.

### K–Ar dating

The sample preparations and K–Ar analyses were conducted at Hiruzen Institute for Geology and Geochronology Co. Ltd. (http://www.geohiruzen.co.jp). The samples were all cut into ~ 0.5 to 1 cm wide slices, crushed with a jaw crusher, and then sieved. Plagioclase or groundmass fractions were separated from several of the resulting size fractions. We selected the fractions with the best combination of abundance and purity for K and Ar analysis (Table S3B). All samples were then cleaned by running water (several times), deionized water (two or three times), and then dried. The samples were then separated using a magnetic separator to separate plagioclase or groundmass from other minerals. The analysis of potassium and argon in the separated samples, and calculations of ages and errors were carried out by the methods of ref.^48^. Potassium was analyzed by flame photometry, using a 2000 ppm Cs buffer, with an analytical error of 2% at a 2σ confidence level. Argon analyses were conducted with a 15 cm radius sector type mass spectrometer with a single collector system, using the isotopic dilution method and an ^38^Ar spike. Multiple runs of the standard (JG-1 biotite, 91 Ma) gave an error of about 1% at a 2σ confidence level^48^.

### U–Pb dating of zircon

Zircon grains for U–Pb geochronology were separated using a high-voltage pulse power fragmentation device (SELFRAG) at the NMNS. The heavy minerals were concentrated by panning and further processed with a hand magnet, and the remaining fractions were purified using heavy liquid (diiodomethane) separation. An adequate amount of zircons (approximately 300 grains) was randomly handpicked from each sample under a binocular stereo microscope and mounted together with TEOMORA-2 (417 Ma; ref.^49^) and OD-3 (33 Ma; ref.^50^) zircon standards and SRM NIST 610 glass standard in an epoxy disc. Sample discs were polished down to the center of the zircon grains, and cathodoluminescence and backscattered electron images were taken using a JEOL JSM-6610 scanning electron microscope (SEM) at the NMNS. The U–Pb–Th isotope dating was conducted using an NWR213 laser ablation system and an Agilent 7700 × ICP–MS at the NMNS (Table S3C). Experimental conditions, measurement procedures, and data reduction followed those of ref.^51^. The weighted mean age of OD-3 analyzed during the analytical sessions was 32.55 ± 0.37 Ma (95% confidence interval, n = 18, MSWD = 1.07), which is concordant with the reported reference age of 33.0 ± 0.1 Ma^50^.

### Major element analyses of minerals

The compositions of (micro) phenocrysts within five volcanic rocks (samples PP04, PP05A, PP06, PP08, PP11) from Popa volcano were obtained using a JEOL JXA-8230 WDS electron probe microanalyzer (EPMA) at the NMNS (Table S4). This analysis used an accelerating voltage of 15 kV, a beam current of 20 nA, and a focused beam of ~ 2 μm diameter for plagioclase, augite, hypersthene, hornblende, and apatite. On the other hand, the accelerating voltage of 20 kV with the same beam current and beam diameter were applied for olivine and magnetite determination to get precise concentrations of NiO and Cr_2_O_3_. Counting times of 15 s were used for major elements (SiO_2_, FeO and MgO in olivine; SiO_2_, FeO, MgO, CaO and Na_2_O in hypersthene and augite; SiO_2_, Al_2_O_3_, FeO, MgO, CaO and Na_2_O in hornblende; SiO_2_, Al_2_O_3_, CaO and Na_2_O in plagioclase; TiO_2_ and FeO in magnetite; and CaO and P_2_O_5_ in apatite) and 60 s for minor elements. All analyses were corrected using an atomic number, absorption, and fluorescence (ZAF) correction.

### Computing a primary melt composition

In order to estimate primary melts for volcanoes in central Myanmar, we conducted inverse modeling that is the same as ref.^21^. We selected 30 Monywa high-MgO (> 10 wt.%) medium-K basalts^1,21^ that would have experienced fractional crystallization of only olivine. One Popa high-MgO (> 10 wt.%) medium-K basalt (sample MV97-18) reported by ref.^20^ was also selected. According to the method of ref.^52^, olivine fractionation was corrected for by incrementally (0.5 wt.%) adding equilibrium olivine back into the melt until the melt is in equilibrium with olivine having forsterite content [Mg/(Mg + Fe^2+^) in molar percent] of 90. Here, we assumed that Fe^3+^ in total iron had a molar ratio of 0.2. By adding 2.5–7.0 wt.% olivine to the medium-K basalts, the primary melt compositions were estimated.

## Supplementary Information

Supplementary Figure S1.

Supplementary Figure S2.

Supplementary Figure S3.

Supplementary Figure S3 continued.

Supplementary Figure S4.

Supplementary Figure S5.

Supplementary Figure S6.

Supplementary Information.

Supplementary Tables.
