# Supplementary figures and images for: Petrogenesis of isotopically enriched Quaternary magma with adakitic affinity associated with subduction of old lithosphere beneath central Myanmar
Source: Sci Rep. 2022 Feb 24;12:3137. doi: 10.1038/s41598-022-07097-4 (PMC8873435; doi:10.1038/s41598-022-07097-4)

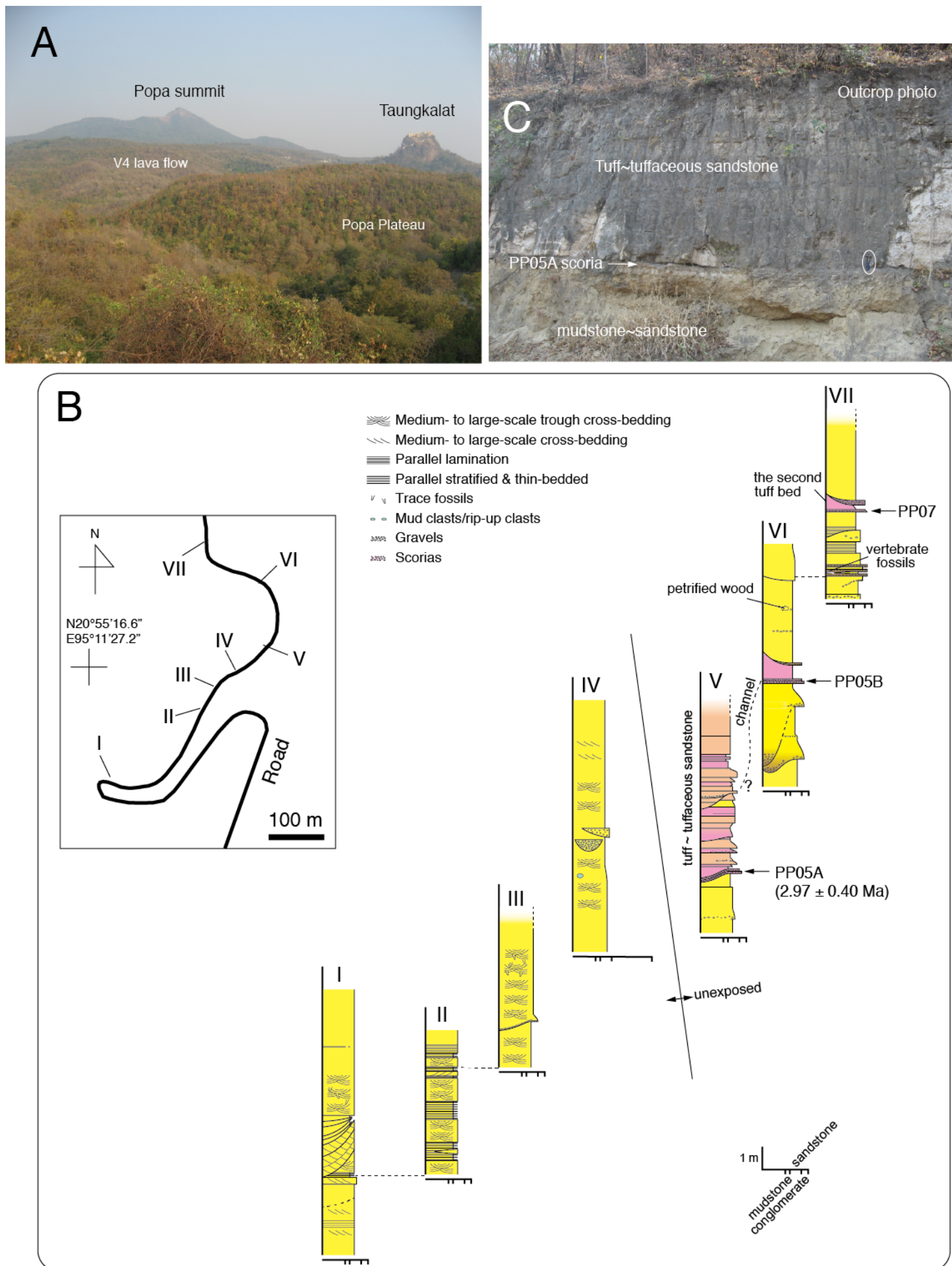

Supplementary Figure S1 (Sano et al.)

Supplement: Supplementary file 1 — Supplementary Figure S1. [file 41598_2022_7097_MOESM1_ESM.pdf]

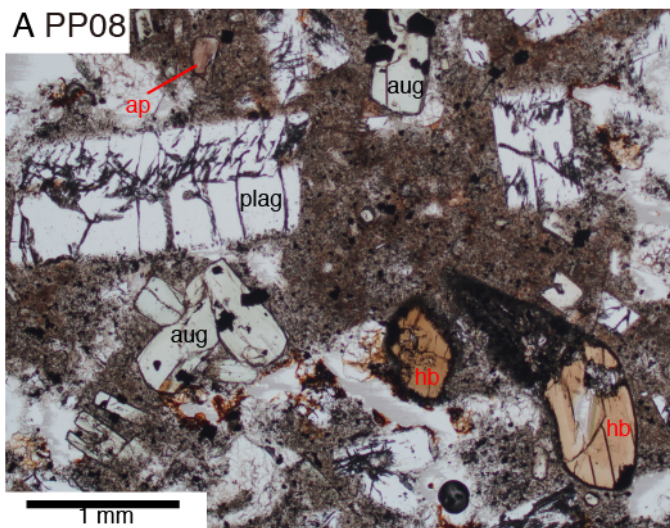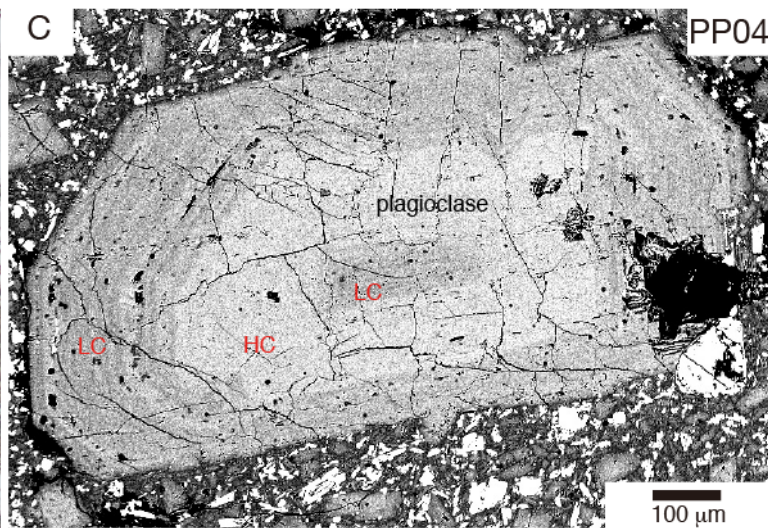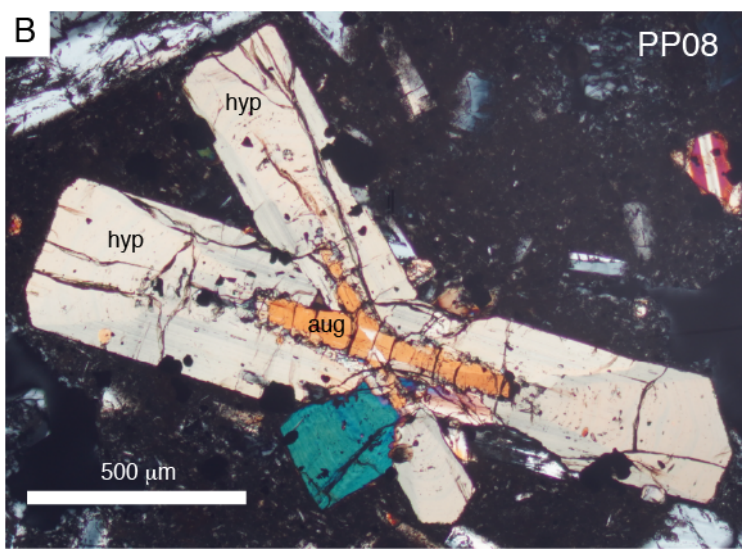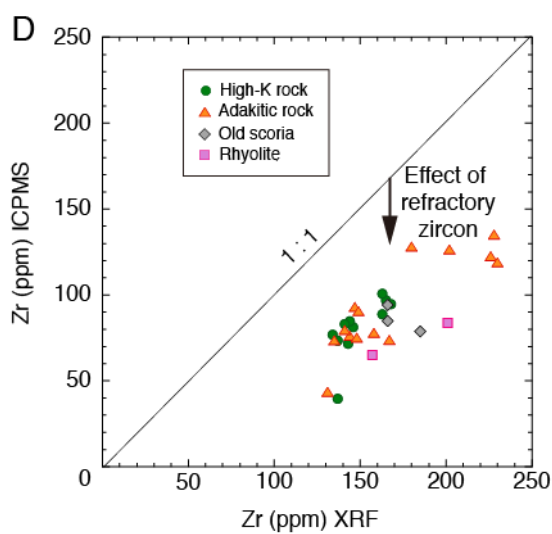

Supplementary Figure S2 (Sano et al.)

Supplement: Supplementary file 2 — Supplementary Figure S2. [file 41598_2022_7097_MOESM2_ESM.pdf]

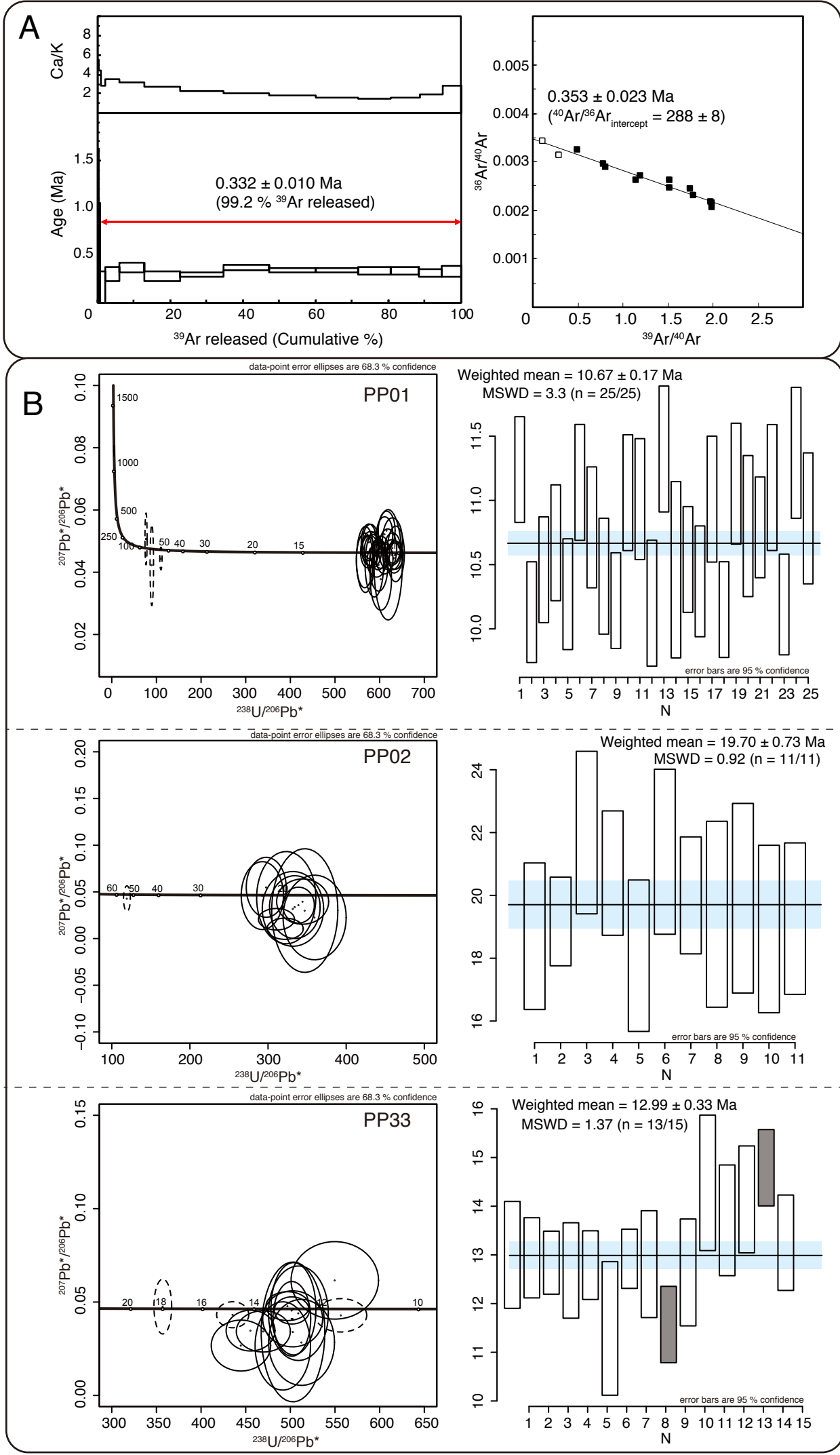

Supplementary Figure S3 (Sano et al.)

Supplement: Supplementary file 3 — Supplementary Figure S3. [file 41598_2022_7097_MOESM3_ESM.pdf]

C

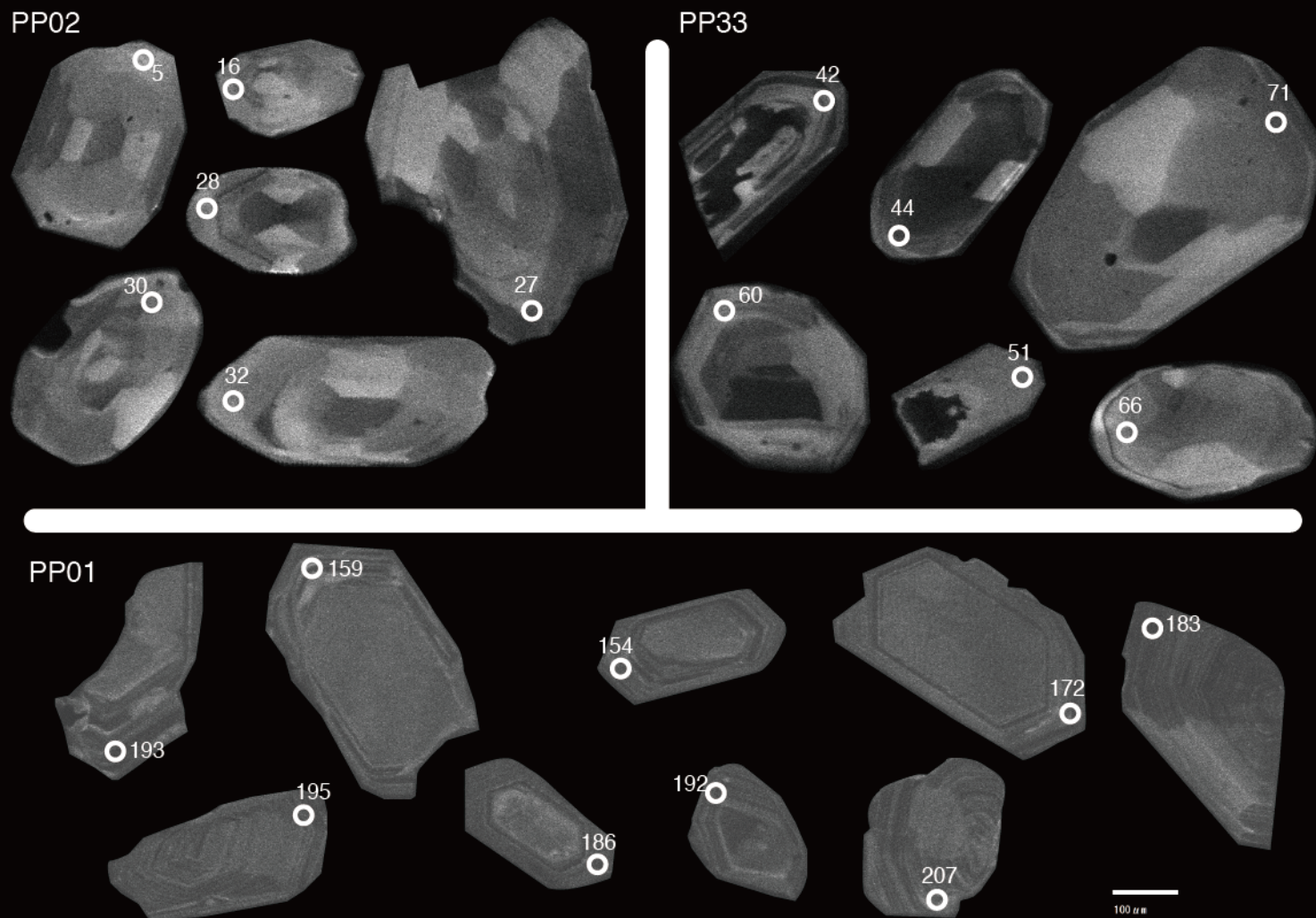

Supplementary Figure S3. continued (Sano et al.)

Supplement: Supplementary file 4 — Supplementary Figure S3 continued. [file 41598_2022_7097_MOESM4_ESM.pdf]

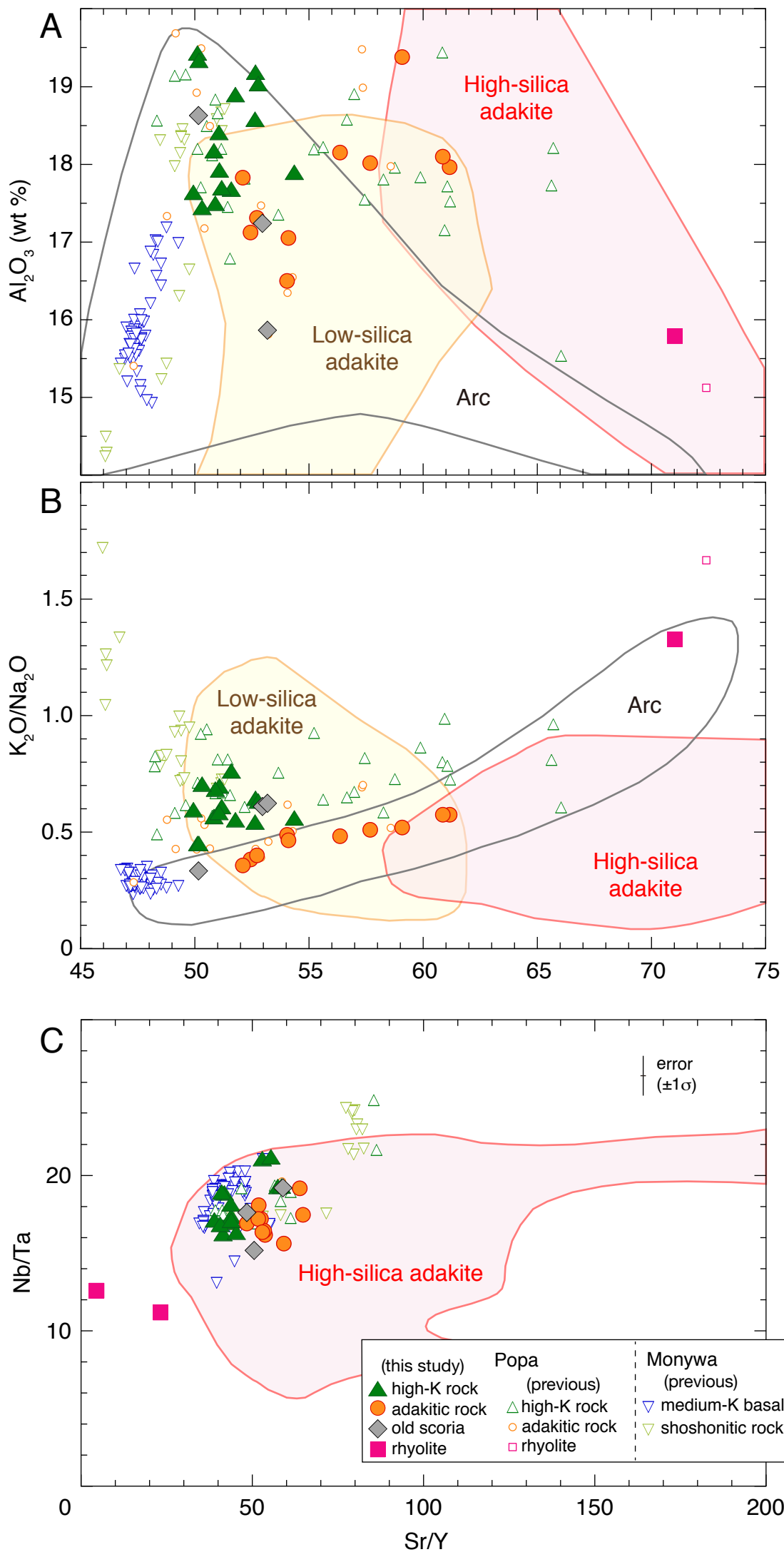

Supplementary  
Figure S4  
(Sano et al.)

Supplement: Supplementary file 5 — Supplementary Figure S4. [file 41598_2022_7097_MOESM5_ESM.pdf]

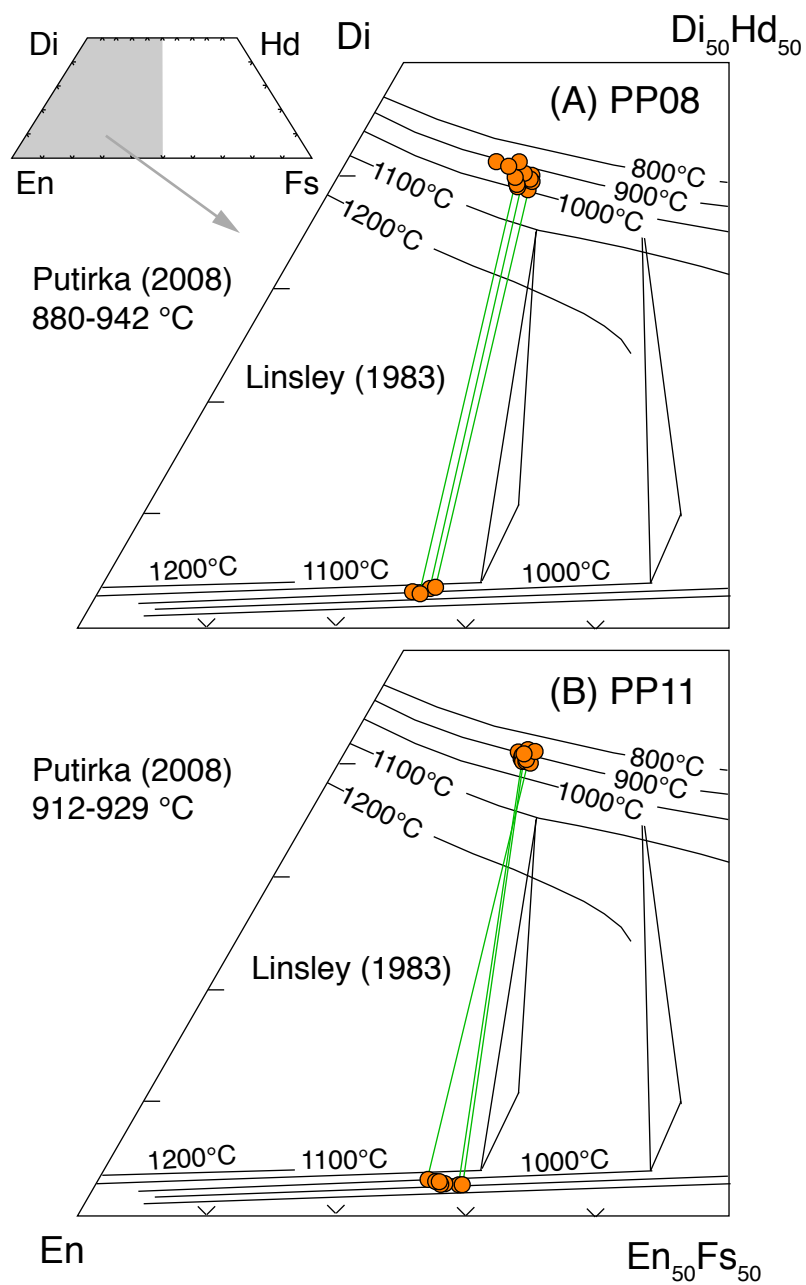

Supplementary Figure S5 (Sano et al.)

Supplement: Supplementary file 6 — Supplementary Figure S5. [file 41598_2022_7097_MOESM6_ESM.pdf]

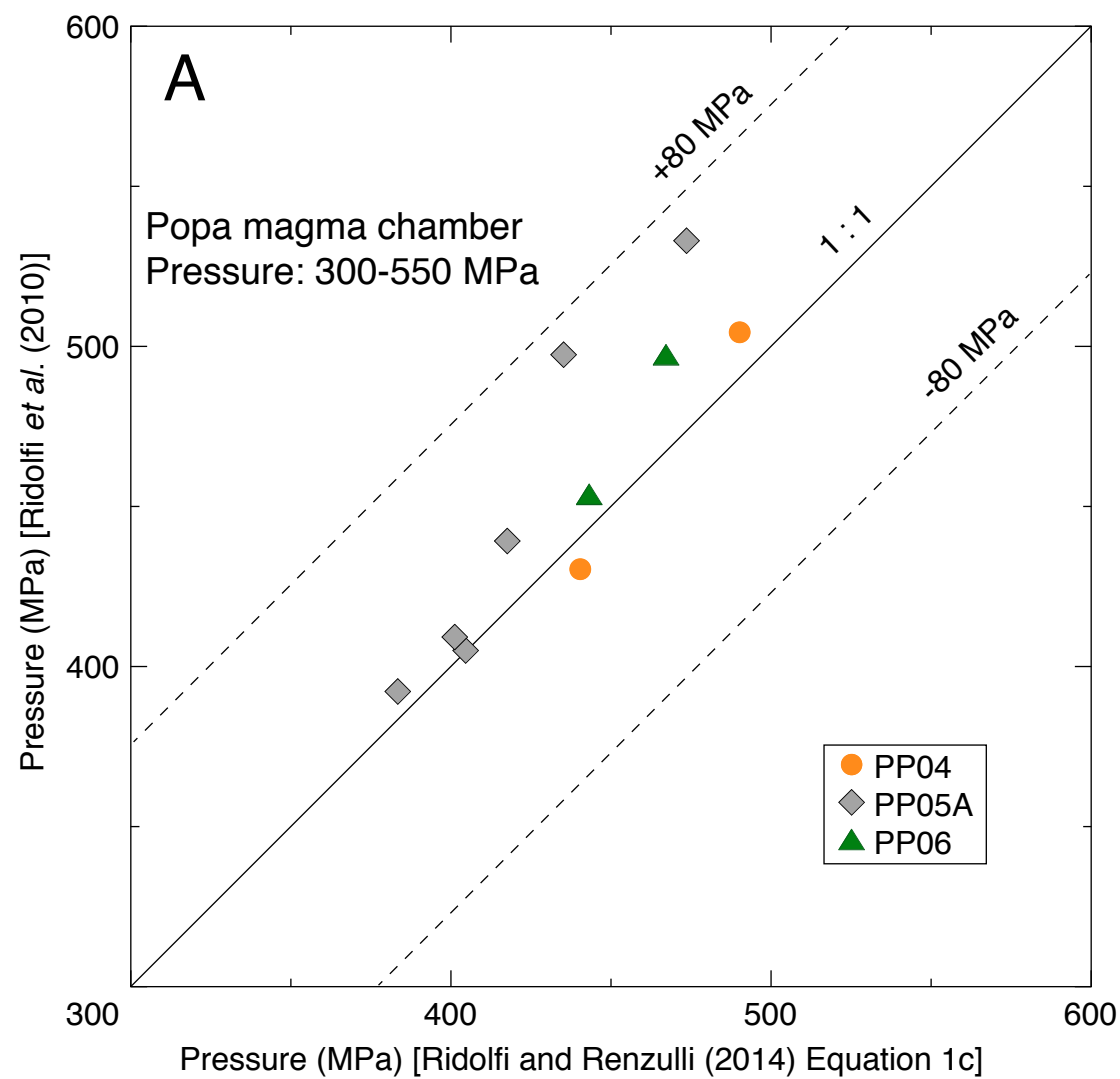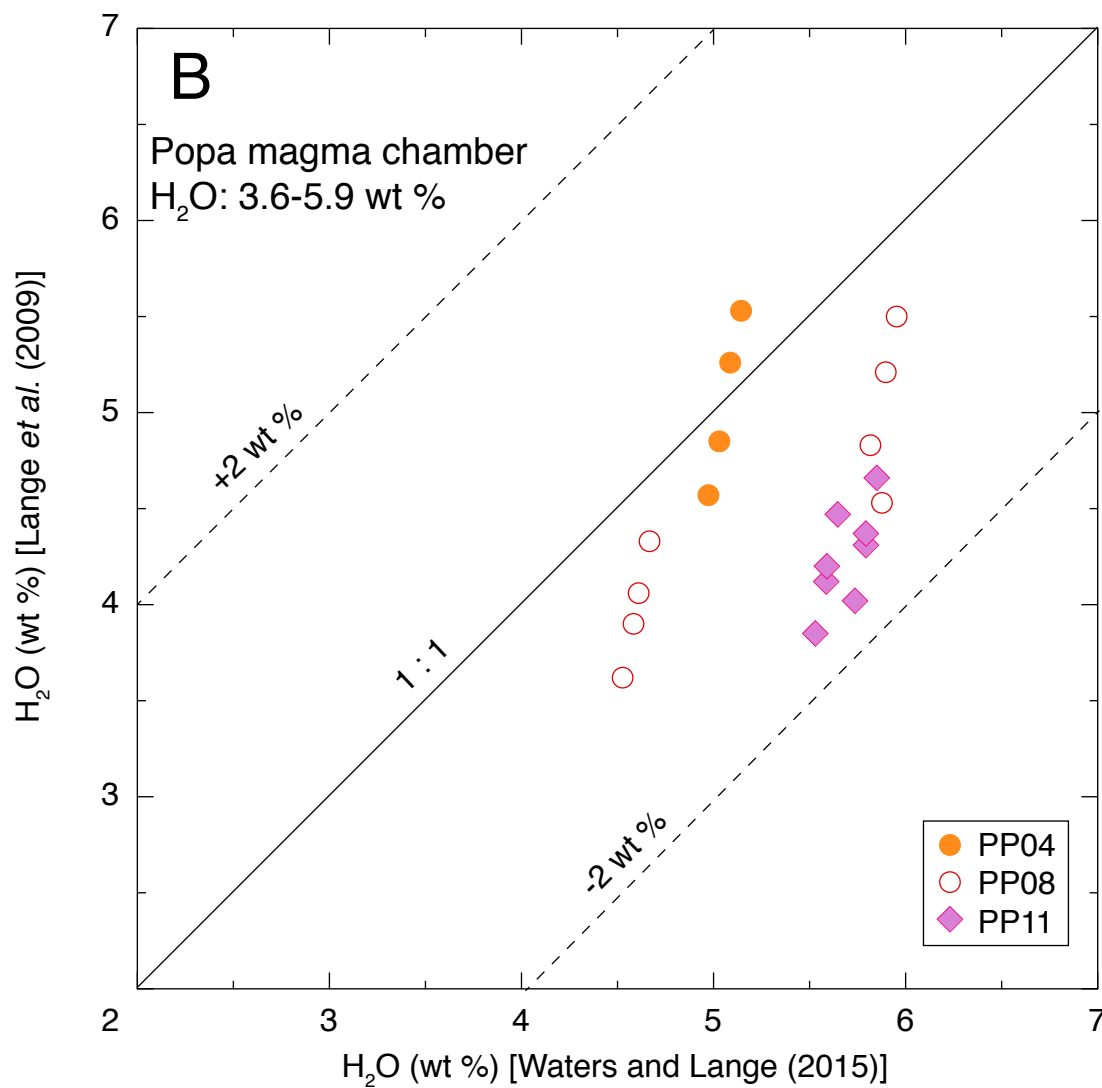

Supplement: Supplementary file 7 — Supplementary Figure S6. [file 41598_2022_7097_MOESM7_ESM.pdf]
