# Supplementary material for: Petrogenesis of isotopically enriched Quaternary magma with adakitic affinity associated with subduction of old lithosphere beneath central Myanmar
Source: Sci Rep. 2022 Feb 24;12:3137. doi: 10.1038/s41598-022-07097-4 (PMC8873435; doi:10.1038/s41598-022-07097-4)
Supplement: Supplementary file 8 — Supplementary Information. [file 41598_2022_7097_MOESM8_ESM.doc]

**Supplementary Information**

**Petrogenesis of isotopically enriched Quaternary magma with adakitic affinity associated with subduction of old lithosphere beneath central Myanmar**

Takashi Sano^1*^, Kenichiro Tani^1^, Shigekazu Yoneda^2^, Hla Min^3^, Thaung Htike^4^, Zin Maung Maung Thein^5^, Osamu Ishizuka^6^, Nao Kusuhashi^7^, Reiko T. Kono^8^, Masanaru Takai^9^, Chris E. Conway^6^

^1^ Department of Geology and Paleontology, National Museum of Nature and Science, 4-1-1 Amakubo, Tsukuba, Ibaraki 305-0005, Japan

^2^ Department of Science and Engineering, National Museum of Nature and Science, 4-1-1 Amakubo, Tsukuba, Ibaraki 305-0005, Japan

^3^ Department of Geology, Meiktila University, Meiktila, Myanmar

^4^ Pro-Rector, University of Magway, Magway, Myanmar

^5^ Department of Geology, University of Mandalay, Mahaaungmyae 05032, Mandalay, Myanmar

^6^ Research Institute of Earthquake and Volcano Geology, AIST, 1-1-1 Higashi, Tsukuba, Ibaraki 305-8567, Japan

^7^ Faculty of Science, Ehime University, 2-5 Bunkyo-cho, Matsuyama, Ehime 790-8577, Japan

^8^ Faculty of Letters, Keio University, Hiyoshi 4-1-1, Yokohama, Kanagawa, 223-8521, Japan

^9^ Primate Research Institute, Kyoto University, Inuyama, Aichi 484-8506, Japan

* Corresponding author. Tel.: +81-29-853-8165; fax: +81-29-853-8998.

*E-mail address*: sano@kahaku.go.jp (T. Sano).

**Supplementary Figures and Figure legends**

**Supplementary Figure 1**. (A) Morphological features of Mt. Popa, its volcanic plug (Taungkalat), Popa Plateau, and V4 lava flow. The photograph was taken from near the location of sample PP06 (in Fig. 1) looking eastward. (B) Sections of Irrawaddy Formation beneath Popa Plateau; locations PP05 and PP07 in Fig. 1. The formation consists of unconsolidated, poorly to thinly bedded sandstones deposited in a fluvial environment (Barber et al., 2017). Samples from three scoria layers (samples PP05A, PP05B, PP07) in the Irrawaddy Formation were collected for our age dating and geochemical analyses. Top-left inset shows locations of the sections. (C) Field occurrence of PP05A scoria layer. A hammer (within a white ellipse) is present to show the scale of the photo.

**Supplementary Figure 2**. (A), (B) Photomicrographs of sample PP08 thin section. (A) is under plane-polarized light and (B) is under cross-polarized light. Abbreviations of minerals are plag, plagioclase; aug, augite; hb, hornblende; ap, apatite; hyp, hypersthene. (C) Back-scattered electron microprobe image of oscillatory zoned plagioclase in PP04 sample. Abbreviations of type of plagioclase core are HC, high-An core; LC, low-An core. (D) Zr concentrations measured by ICP-MS versus those measured by XRF. Note that Zr concentrations by ICP-MS are distinctly lower than those by XRF for all samples, indicating that refractory zircon minerals are present in the sample solutions for ICP-MS.

**Supplementary Figure 3**. (A) Age plateau and Ca/K plots (left) and inverse isochron diagram (right) for ^40^Ar/^39^Ar incremental heating analysis of groundmass sample separated from PP19 basalt from Popa volcano. All errors for ^40^Ar/^39^Ar results are reported at one standard deviation. Errors for ages include analytical uncertainties for Ar isotope analysis, correction for interfering isotopes and J value estimation. An error of 0.5% was assigned to J values as a pooled estimate during the course of this study. Results of Ar isotopic analyses and correction factors for interfering isotopes are presented in Table S3A. The plateau age was calculated as weighted means of ages of plateau-forming steps, where each age was weighted by the inverse of its variance. The age plateau was determined following the definition by Fleck et al. (1977). The inverse isochron was calculated using York’s least-squares fit, which accommodates errors in both ratios and correlations of errors (York, 1969). (B) Zircon U-Pb analysis. Left panels: Terra-Wasserburg concordia diagrams for analyzed zircons. Error ellipses represent 68.3% confidence intervals. Solid ellipses, analyses used in weighted mean age calculations; dotted ellipses, excluded analyses. ^207^Pb* and ^206^Pb* indicate radiometric ^207^Pb and ^206^Pb, respectively. Right panels: Age distribution plots. Quoted ages are weighted means of ^207^Pb-corrected ^206^Pb/^238^U spot ages (n analyses, errors at 95% confidence intervals). Gray bars, analyses excluded from weighted mean age calculations. Corrections for common Pb on the concordia diagrams were carried out using ^208^Pb-correction method (Williams, 1998) and ^207^Pb-corrected ^206^Pb/^238^U ages were used to calculate weighted means (Williams, 1998). The concordia diagrams were produced and weighted means were calculated using the IsoplotR program (Vermeesch, 2018) Outliers excluded from mean age calculations are indicated in Table S3C, and are selected from statistical variation using a modified version of Chauvenet's criterion in the IsoplotR program. (C) Representative cathodoluminescence images of analyzed zircons. Analysis spots are marked with white ellipses, and labeled according to spot number, as listed in Supplementary Table S3C.

**Supplementary Figure 4.** (A) Al_2_O_3_, (B) K_2_O/Na_2_O versus SiO_2_, and (C) Nb/Ta versus Sr/Y diagrams for the Quaternary volcanic rocks in central Myanmar. References for previous data for Popa and Monywa volcanoes are from Stephenson and Marshall (1984), Maury et al. (2004), Lee et al. (2016), Belousov et al. (2018), and Zhang et al. (2020). Low-silica adakite and high-silica adakite fields are from Moen (2009). In (A) and (B), the two standard deviations of repeated sample analyses are less than the size of the symbols (Table S2). In (C), an error bar is ±1 sigma of repeated sample analyses (Table S2).

**Supplementary Figure 5**. Pyroxene compositions for PP08 and PP11 adakitic rocks, with temperatures of crystallization for the Popa magma estimated using the two-pyroxene geothermometer (Lindsley, 1983). Coexisting augite and hypersthene (Table S4) are connected by green lines. The two sigma uncertainties associated with the counting statistics for the electron microprobe analyses are smaller than the size of the symbols. Calculated temperatures based on another two-pyroxene geothermometer (Table S4; Putirka, 2008) are also noted.

**Supplementary Figure 6**. (A) Predicted pressures calculated using the geobarometer of Ridolfi et al. (2010) versus those of Ridolfi and Renzulli (2012). The calculated pressures by the two different geobarometers are the same within ±80 MPa error. When we consider the error and the calculated results in Table S4, crystallization pressure is estimated to be 300–550 MPa. (B) Predicted H_2_O contents calculated using the hydrometer of Lange et al. (2009) versus those of Waters and Lange (2015). Considering the calculated results of the two hydrometers (Table S4), H_2_O content of adakitic magma is estimated to be 3.6–5.9 wt %.

**Supplementary Text**

**1. Chemical separation of Sr and Nd**

Chemical separation of Sr and Nd were conducted at NMNS following the methods used by Pin et al. (1994, 1997), Noguchi et al. (2011), and Kuritani and Nakagawa (2016).

Firstly, the sample was acid-leached. Approximately 100 mg of the rock powder was placed into a 5ml PFA jar and ~2.5 ml of 6M HCl (EL-grade, Kanto Chemical Co. Inc., Tokyo) was added. Teflon PFA jar (Savillex Corp.) was sealed with screw cap and heated at 120 °C on a hot plate for 1 hour. After the jar was cold, the dissolved sample was centrifuged at 3000 rpm for 3 minutes, and supernatant solution was sucked and thrown away. Then, ~5 ml Milli-Q water was added, sealed with screw cap, and shaken well by hand. Again, the sample was centrifuged at 3000 rpm for 3 minutes, and the supernatant solution was sucked and thrown away.

Next, the residue was dissolved by using acid. After 1 ml 12 M HNO_3_ (EL-grade, Kanto Chemical Co. Inc.), 0.5 ml 70% HClO_4_ (Tamapure AA-100 grade, Tama Chemicals, Co. Ltd.), 1 ml 38% HF (Tamapure AA-100 grade, Tama Chemicals, CO. Ltd.) were added to the acid-leached rock powder, the PFA jar was sealed with screw cap and heated at 120 °C on a hot plate for 1 hour. Then, the PFA jar was opened and the mixture was evaporated by step heating at progressively increasing temperature: 120°C (>8 hours); 140 °C (>8 hours); 160 °C (>8 hours); 180 °C (>8 hours). The residue was then dissolved with 1-2 ml 6M HCl (EL-grade, Kanto Chemical Co. Inc.), sealed with screw cap, and heated at 120 °C on a hot plate for 1 hour. Again, the PFA jar was opened and the solution was evaporated at 120 °C on a hot plate for 2-4 hours. Finally, the residue was dissolved with 0.5 ml 2M HNO_3_, sealed with screw cap, and heated once again at 120 °C on a hot plate for 10-20 minutes.

Strontium separation was carried out using a shrink-fit Teflon column filled with ~0.1 ml of the Sr-Spec resin (50-100 mm particle size; Eichrom Technologies, Inc.). The column was washed with 1 ml of Milli-Q water and conditioned with 0.5 ml of 2M HNO_3_. The sample solution dissolved in 0.5 ml 2M HNO_3_ was centrifuged at 3000 rpm for 20 minutes, and only clear upper portion of the solution was loaded on the column and waited until it was dropped. The dropped solution was kept to separate Nd (see below). The column was then washed with 1.2 ml 8M HNO_3_ to remove unwanted cations. After the column was treated with 0.4 ml 2M HNO_3_, Sr was stripped and collected with 1 ml of 0.05 M HNO_3_. Then, the Sr solution was passed through another shrink-fit Teflon column filled with ~0.1 ml of pre-filter resin (100-150 mm particle size; Eichrom Technologies, Inc.) to remove organic matter. The final solution contained ~90 % Sr. Lastly, a suitable amount of solution (Sr ~200 ng) was evaporated to dryness at 80 °C on a hot plate for 2-4 hours and considered ready for mass spectrometry.

Neodymium was separated from the dropped solution (see above) by two-step column separation method. The first column was a shrink-fit Teflon column filled with ~0.1 ml of the transuranic-element specific (TRU Spec) resin (50-100 mm particle size; Eichrom Technologies, Inc.) to separate light rare-earth elements (LREEs) from the solution. The column was washed with 1 ml of Milli-Q water and conditioned with 0.5 ml of 1M HNO_3_. Approximately 0.5 ml of 10 wt % Ascorbic acid (Kanto Chemical Co. Inc.) was added to avoid oxidation of the solution, and then, loaded on the column and waited until the solution was dropped. The column was then washed with 1 ml 1M HNO_3_ to remove unwanted cations. The LREEs were stripped and collected with 1 ml of 0.05 M HNO_3_. The collected solution was evaporated to dryness at 80 °C on a hot plate for 12 hours and the residue was used for the following second column separation. The second column was a polypropylene column (5 mm f: Muromachi Mini-column S, Muromachi Chemicals Inc.). Approximately 1 ml of Ln Spec resin (50-100 mm particle size; Eichrom Technologies, Inc.) was loaded into the polypropylene column and the resin top was covered with a porous 30 mm polyethylene filter to prevent the flotation of the resin. The column was washed with 9 ml of Milli-Q water and conditioned with 4 ml of 0.2 M HCl. The residue sample after the dryness was dissolved with 0.1 ml 0.2 M HCl and then loaded on the column. The Ln Spec column was rinsed with 1.9 ml and 6.0 ml of 0.2 M HCl, and separation of individual LREE from La to Nd was done. Nd was stripped and collected with 5.5 ml of 0.2 M HCl. Then, Nd solution was passed through another polypropylene column filled with ~0.2 ml of pre-filter resin (100-150 mm particle size; Eichrom Technologies, Inc.) to remove organic matter. The final fraction contained 50-60 % Nd, 40-50 % Pr, <2% Ce, and no detectable Sm. Lastly, the fraction was evaporated to dryness at 100 °C on a hot plate for 12-15 hours and considered ready for mass spectrometry. The maximum blank was 5 pg for Sr and 0.5 pg for Nd.

**2. Additional explanations for Figures 2, 3 and S4**

In Fig. 2A, the classification of volcanic rock types and the fields for subalkalic and alkalic are from Le Maitre (2002) and Miyashiro (1978), respectively. In Figs. 2A, E, 3A, B and Supplementary Fig. S4A–C, low-silica adakite, high-silica adakite, and normal arc fields are from Moen (2009). In Fig. 2B, the fields for shoshonite, high-K, medium-K, and low-K are from Peccerillo and Taylor (1976). In Fig. 2C, the fields for tholeiitic and calc-alkaline are from Miyashiro (1974). In Fig. 3D, subscript t denotes the initial isotope composition at the time when the magma crystallized. Data fields of worldwide Quaternary (and two Miocene) adakites and adakitic rocks are classified into two groups based on their proposed magma origins; one is slab melting (red), and another is high pressure crystallization from basaltic magma and/or melting of slab-melt modified mantle (blue). Modern (0 Ma) and 200 Ma CIR mid-ocean ridge basalt (MORB) data are from Ito et al. (1987) and Rehkämper and Hofmann (1997), with 200 Ma CIR magma source field corrected for radiogenic ingrowth assuming parent-daughter rations of MORB source mantle (Salter and Stracke, 2004). Mixing endmembers of the black curved line are a pelagic sediment (sample V28-343) reported by Gasparson and Varne (1998) [Sr, 160 ppm; Nd, 31.9 ppm; ^87^Sr/^86^Sr, 0.71162; ^144^Nd/^143^Nd, 0.512278] and a CIR magma source [Sr, 9.8 ppm; Nd, 0.713 ppm; ^87^Sr/^86^Sr, 0.7028; ^144^Nd/^143^Nd, 0.513080]. Data fields for Quaternary adakites and adakitic rocks are from previous reports: Aleutian (Yogodzinski et al., 1993, 1995); Solandar, NZ (Foley et al., 2013); St. Helens (Leeman et al., 1990); Panama-Costa Rica (Gazel et al., 2011); Ecuador (Bourdon et al., 2003); SW Japan (Feineman et al., 2013; Pineda-Velasco et al., 2018; Yamamoto and Hoang, 2019); Camiguin (Castillo et al., 1999); Mindanao (Macpherson et al., 2006); Central Japan (Nakamura and Iwamori, 2013). Data fields for Miocene adakitic rocks near the study area (central Myanmar) are also shown: North Myanmar (Li et al., 2019); South Tibet (Gao et al., 2010). The North Myanmar adakitic rocks were collected from Shangalon area (top-left inset of Fig. 1). The data field of Old Indian/Neo-Tethyan MORB is constrained by using only data of glass samples from old western Indian MORB (Mahoney et al., 1998) and basalts from Indus-Yarlung Suture (Zhang et al., 2005). Data from Xigaze Neo-Tethyan ophiolites (Xu and Castillo, 2004) are also included in the field.

**References (supplemental information)**

Bacon C. R. & Dnntt, T. H. Compositional evolution of the zoned calcalkaline magma chamber of Mount Mazama, Crater Lake, Oregon. *Contrib. Mineral. Petrol*. **98**, 224–256 (1988).

Barber, A. J., Khin Zaw & Crow, M. J., eds. Myanmar Geology, Resources, and Tectonics. *Geological Society, London, Memoirs* **48**, 773p (the Geological Society, 2017).

Belousov, A. *et al*. Holocene eruptions of Mt. Popa, Myanmar: Volcanological evidence of the ongoing subduction of Indian Plate along Arakan Trench. *J. Volcanol. Geotherm. Res*. **360**, 126–138 (2018).

Bourdon, E. *et al*. Magmatic response to early aseismic ridge subduction: the Ecuadorian margin case (South America). *Earth Planet. Sci. Lett*. **205**, 123–13 (2003).

Bouvier, A., Vervoort, J. D. & Patchett, P. J. The Lu-Hf and Sm-Nd isotopic composition of CHUR: constraints from unequilibrated chondrites and implications for the bulk composition of terrestrial planets. *Earth Planet. Sci. Lett*. **273**, 48–57 (2008).

Castillo, P. R., Janney, P. E. & Solidum, R. U. Petrology and geochemistry of Camiguin Island, southern Philippines: insights to the source of adakites and other lavas in a complex arc setting. *Contrib. Mineral. Petrol*. **134**, 33–51 (1999).

Defant, M. J. & Drummond, M. S. Derivation of some modern arc magmas by melting of young subducted lithosphere. *Nature* **347**, 662–665 (1990).

DePaolo, D. J. Trace element and isotopic effects of combined wallrock assimilation and fractional crystallization. *Earth Planet. Sci. Lett*. **53**, 189–202 (1981).

Feineman, M. *et al*. Sediment-enriched adakitic magmas from the Daisen volcanic field, Southwest Japan. *Geochem. Geophys. Geosyst*. **14**, <https://doi.org/10.1002/ggge.20176> (2013).

Fleck, R. J., Sutter, J. F. & Elliot, D. H. Interpretation of discordant ^40^Ar/^39^Ar age-spectra of Mesozoic tholeiites from Antarctica. *Geochim. Cosmochim. Acta* **41**, 15–32 (1977).

Foley, F. V. *et al*. Magmatic Evolution and Magma Mixing of Quaternary Adakites at Solander and Little Solander Islands, New Zealand. *J. Petrol*. **54**, 703–744 (2013).

Fourny, A., Weis, D., & Scoates, J. S. Comprehensive Pb-Sr-Nd-Hf isotopic, trace element, and mineralogical characterization of mafic to ultramafic rock reference materials. *Geochemistry Geophysics Geosystems* **17**, 739–773 (2016).

Gao, Y. *et al*. Adakitic rocks from slab melt-modified mantle sources in the continental collision zone of southern Tibet. *Lithos* **119**, 651-663 (2010).

Gasparson, M. & Varne, R. Crustal assimilation versus subducted sediment input in west Sunda arc volcanics: an evaluation. *Mineral. Petrol*. **64**, 89–117 (1998).

Gazel, E. *et al*. Plume-subduction interaction in southern Central America: Mantle upwelling and slab melting. *Lithos* **121**, 117–134 (2011).

Irving, A. J. A review of experimental studies of crystal/liquid trace element partitioning. *Geochim. Cosmochim. Acta* **42**, 743–4527 (1978).

Ito, E., White, W. M. & Göpel, C. The O, Sr, Nd and Pb isotope geochemistry of MORB. *Chem. Geol*. **62**, 157–176 (1987).

Kelemen, P. B., Yogodzinski, G. M. & Scholl, D. W. Along-strike variation in the Aleutian island arc: Genesis of high Mg# andesite and implications for continental crust (ed. Eiler, J.) *Inside the Subduction Factory. AGU Geophys. Monogr*. **138**, 223–276 (2003).

Kuritani, T. & Nakagawa, M. Origin of ultra rear-arc magmatism at Rishiri Volcano, Kuril Arc. *Geochem. Geophys. Geosyst*. **17**, 4032-4050 (2016).

Lange, R. A., Frey, H. M. & Hector, J. A thermodynamic model for the plagioclase-liquid hygrometer/thermometer. *Am. Mineral*. **94**, 494–506 (2009).

Lee, H. -Y., Chung, S. -L. & Yang, H. -M. Late Cenozoic volcanism in central Myamar: Geochemical characteristics and geodynamic significance. *Lithos* **245**, 174–190 (2016).

Leeman, W. P. *et al*. Compositional Diversity of Late Cenozoic Basalts in a Transect Across the Southern Washington Cascades: Implications for Subduction Zone Magmatism. *J. Geophys. Res. Solid Earth* **95**, 19,561–19,582 (1990).

Le Maitre, R. W., ed. *Igneous Rocks: A Classification and Glossary of Terms, 2nd edition.* 236 p (Cambridge University, 2002).

Li, J. -X. *et al*. Subduction of Indian continental lithosphere constrained by Eocene-Oligocene magmatism in northern Myanmar. *Lithos* **348-349**, 105211 (2019).

Lindsley, D. H. Pyroxene thermometry. *Am. Mineral*. **68**, 477–493 (1983).

Macpherson, C. G., Dreher, S. T. & Thirlwall, M. F. Adakites without slab melting: High pressure differentiation of island arc magma, Mindanao, the Philippines. *Earth Planet. Sci. Lett*. **243**, 581–593 (2006).

Mahoney, J. J. *et al*. Tracing the Indian ocean mantle domain through time: isotopic results from old west Indian, east Tethyan, and south Pacific seafloor. *J. Petrol*. **39**, 1285–1306 (1998).

Martin, H. Adakitic magmas: modern analogues of Archaean granitoids. *Lithos* **45**, 411–429 (1999).

Martin, H. *et al*. An overview of adakite, tonalite-tronhjemite-granodiorite (TTG), and sanukitoid: relationships and some implications for crustal evolution. *Lithos* **79**, 1–24 (2005).

Maury, R. C. *et al*. Fusion de la croute océanique dans les zones de subduction/collision récenters: l’exemple de Mondanao (Philippines). *Bull. Soc. Geol. France* **167**, 579–595 (1996).

Maury, R. C. *et al*. Quaternary calc-alkaline and alkaline volcanism in an hyperoblique convergence setting, central Myanmar and western Yunnan. *Bull. Soc. Geol. France* **175**, 461–472 (2004).

Miyashiro, A. Volcanic rock series in island arc and active continental margins. *Am. J. Sci*. **274**, 321–355 (1974).

Miyashiro, A. Nature of volcanic alkalic rock series: *Contrib. Mineral. Petrol*. **66**, 91–104 (1978).

Moen, J. -F. High Sr/Y and La/Yb rations: The meaning of the “adakitic signature”. *Lithos* **112**, 556–574 (2009).

Nakamura, H. & Iwamori, H. Generation of adakites in a cold subduction zone due to double subduction plates. *Contrib. Mineral. Petrol*. **165**, 1107–1134 (2013).

Noguchi, T. *et al*. Barite geochemistry from hydrothermal chimneys of the Okinawa Trough: insight into chimney formation and fluid/sediment interaction. *J. Mineral. Petrol. Sci*. **106**, 26–35 (2011).

Peccerillo, A. & Taylor, S. R. Geochemistry of Eocene calc-alkaline volcanic rocks from the Kastamonu area, northern Turkey. *Contrib. Mineral. Petrol.* **58**, 63–81 (1976).

Pin, C., Francisco, J. & Zalduegui, S. Sequential separation of light rare-earth elements, thorium and uranium by miniaturized extraction chromatography: Application to isotopic analyses of silicate rocks. *Anal. Chimica Acta* **339**, 79–89 (1997).

Pin, C., Briot, D., Bassin, C. & Poitrasson, F. Concomitant separation of strontium and samarium–neodymium for isotopic analysis in silicate samples, based on specific extraction chromatography. *Anal. Chimica Acta* **298**, 209–217 (1994).

Pineda-Velasco, *et al*. Production of High-Sr Andesite and Dacite Magmas by Melting of Subducting Oceanic Lithosphere at Propagating Slab Tears. *J. Geophys. Res. Solid Earth* **123**, 3698–3728 (2018).

Prowatke, S. & Klemme, S. Trace element partitioning between apatite and silicate melts: *Geochim. Cosmochim. Acta* **70**, 4513–4527 (2006).

Putirka, K. Thermometers and barometers for volcanic systems. *Rev. Mineral. Petrol*. **69**, 61–120 (2008).

Rehkämper, M. & Hofmann, A. W. Recycled ocean crust and sediment in Indian ocean MORB: *Earth Planet. Sci. Lett*. **147**, 93–106 (1997).

Ridolfi, F. & Renzulli, A. Calcic amphiboles in calc-alkaline and alkaline magmas: thermobarometric and chemometric empirical equations valid up to 1,130 °C and 2.2 GPa. *Contrib. Mineral. Petrol.***163**, 877–895 (2012).

Ridolfi, F., Renzulli, A. & Puerini, M. Stability and chemical equilibrium of amphibole in calc-alkaline magmas: An overview, new thermobarometric formulations and application to subduction-related volcanoes. *Contrib. Mineral. Petrol*. **160**, 45–66 (2010).

Salters, V. J. M. & Stracke, A. Composition of the depleted mantle. *Geochem. Geophys. Geosyst*. **5**, Q05004 (2004).

Sen, C. & Dunn, T. Dehydration melting of a basaltic composition amphibolite at 1.5 and 2.0 GPa: implications for the origin of adakites. *Contrib. Mineral. Petrol*. **117**, 394–409 (1994).

Steiger, R. & Jäger, E. Subcommission on geochronology: convention on the use of decay constants in geo- and cosmochronology. *Earth Planet. Sci. Lett*. **36**, 359–362 (1977).

Stephenson, D. & Marshall, T. R. The petrology and mineralogy of Mt. Popa volcano and the nature of the late-Cenozoic Burma volcanic arc. *J. Geol. Soc. Lond.* **141**, 747–762 (1984).

Vermeesch, P. IsoplotR: a free and open toolbox for geochronology. *Geosci. Front*. **9**, 1479-1493 (2018).

Waters, L. E. & Lange, R. A. An updated calibration of the plagioclase-liquid hygrometer-thermometer applicable to basalts through rhyolites. *Am. Mineral*. **100**, 2172–2184 (2015).

Williams, I. S. U-Th-Pb geochronology by ion microprobe. *Rev. Econom. Geol*. **7**, 1–35 (1998).

Xu, J. -F. & Castillo P. R. Geochemical and Nd-Pb isotope characteristics of the Tethyan asthenosphere: Implications for the origin of the Indian Ocean mantle domain. *Tectonophys*. **393**, 9–27 (2004).

Yamamoto, T. & Hoang, N. Geochemical variations of the Quaternary Daisen adakites, Southwest Japan, controlled by magma production rate. *Lithos* **350-351**, 105214 (2019).

Yogodzinski, G. M., Rubenstone, J. L., Kay, S. M., & Kay, R. W. Magmatic and tectonic development of the Western Aleutians: An oceanic arc in a strike-slip setting. *J. Geophys. Res. Solid Earth* **98**, 11,807–11,834 (1993).

Yogodzinski, G. M. *et al*. Magnesian andesite in the western Aleutian Komandorsky region: Implications for slab melting and processes in the mantle wedge. *Geol. Soc. Am. Bull*. **107**, 505–519 (1995).

York, D. Least squares fitting of a straight line with correlated errors. *Earth Planet. Sci. Lett*. **5**, 320–324 (1969).

Zhang, L. Y. *et al*. Quaternary volcanism in Myanmar: A record of Indian slab tearing in a transition zone from oceanic to continental subduction. *Geochem. Geophys. Geosyst*. **21**, <https://doi.org/10.1029/2020GC009091> (2020).

Zhang, S. -Q. *et al*. Evidence for a widespread Tethyan upper mantle with Indian-ocean-type isotopic characteristics. *J. Petrol*. **46**, 829–853 (2005).
